# A Review of the Current State of Global Surgical Oncology and the Role of Surgeons Who Treat Cancer: Our Profession’s Imperative to Act Upon a Worldwide Crisis in Evolution

**DOI:** 10.1245/s10434-023-13352-3

**Published:** 2023-03-27

**Authors:** Aaron C. Saunders, Miriam Mutebi, T. Subramanyeshwar Rao

**Affiliations:** 1 Summit Cancer Center, Spokane, WA USA; 2 Aga Khan University, Nairobi, Kenya; 3 Basavatarakam Indo-American Cancer Hospital and Research Institute, Hyderabad, India

## Abstract

Worldwide, the capacity of healthcare systems and physician workforce is woefully inadequate for the surgical treatment of cancer. With major projected increases in the global burden of neoplastic disease, this inadequacy is expected to worsen, and interventions to increase the workforce of surgeons who treat cancer and strengthen the necessary supporting infrastructure, equipment, staffing, financial and information systems are urgently called for to prevent this inadequacy from deepening. These efforts must also occur in the context of broader healthcare systems strengthening and cancer control plans, including prevention, screening, early detection, safe and effective treatment, surveillance, and palliation. The cost of these interventions should be considered a critical investment in healthcare systems strengthening that will contribute to improvement in the public and economic health of nations. Failure to act should be seen as a missed opportunity, at the cost of lives and delayed economic growth and development. Surgeons who treat cancer must engage with a diverse array of stakeholders in efforts to address this critical need and are indispensably positioned to participate in collaborative approaches to influence these efforts through research, advocacy, training, and initiatives for sustainable development and overall systems strengthening.

The global burden of disease attributable to cancer is rapidly rising, both in proportional and absolute terms.^1,2^ Worldwide, healthcare systems are inadequately prepared to treat this burden of disease and should be expected to lose ground in terms of relative adequacy of capacity without decisive intervention.^3–9^ Cancer is a neglected element in the public health planning of many of the regions where its threat is most dramatically increasing, and surgical capacity is a neglected element of healthcare systems in those regions.^3–10^ As a result, current ability to surgically treat cancer is critically deficient, and this deficiency will worsen unless a concerted effort is taken to correct it.^6,8^ Now is the time to collectively engage in research, advocacy, training, and initiatives for sustainable development and overall systems strengthening so that we as a profession of surgeons who treat cancer (SWTC) are not increasingly overwhelmed by the needs of the global patient population requiring our care and forced to observe current intra- and international disparities deepen.

## In Humanity’s Fight Against Cancer, is Cancer Winning?

The theory of ‘epidemiologic transition’ has been refined for more than 50 years within the disciplines of public health and demography. While it has its criticisms, it is instructive as a backdrop to discussing the current, pressing needs of global surgical oncology. The theory observes that as the contribution of infectious diseases to population mortality rates declines, there is also an observed increase in life expectancy and an increase in the proportion of mortality attributed to non-communicable diseases (NCDs), including cardiovascular disease and cancer, among others. This is often observed in association with a ‘demographic transition’, in which a decline in birth rate along with the above factors combine to result in an aging population.^1^ From the earliest descriptions, it has been observed that while Western Europe and other geographic areas that are now described as high-income countries (HICs) exhibited epidemiologic transition over a period of more than 150 years, more rapid changes have subsequently been observed in various parts of the world, often associated with more rapid economic, industrial, and societal changes, and tied to more organized public health interventions against infectious disease and other causes of youthful mortality.^1,11,12^ It is observed that as individuals live longer, they are more likely to get cancer; more people worldwide are living long enough, and the increase in the burden of neoplastic disease is dramatic.^2,13^ High rates of risk factor exposure in low- and middle-income countries (LMICs), including tobacco, alcohol, and others, further exacerbate this trend.^1^

Cancer is now the second leading cause of death worldwide after cardiovascular disease and is a rising threat to population health as the global rates of cancer incidence and mortality continue to climb.^2,13,14^ According to data compiled in the Global Cancer Observatory, the estimated global number of new cancer cases in 2020 was almost 19.3 million, nearly doubling from 10 million in the year 2000.^2,15^ The number of global cancer mortalities reported in 2020 approached 10 million deaths, an increase from approximately 6 million deaths in the year 2000.^2,15^ The global number of new cancer cases is projected to increase to 30.2 million cases in 2040, associated with 16.3 million deaths.^2^ The majority of the continued increase in the cancer disease burden is expected to occur in LMICs, consistent with an understanding of the geography where epidemiologic transition is most actively occurring.^7,12^

It should be noted that projections of future burden of neoplastic disease have historically been underestimations; in 2001, the projection for 2020 based on similar data sources was 15 million new cases, now thought to be an underestimation of more than 46% compared with the true increase over that interval, although the projected number of deaths appears to have been more accurate.^2,15^ The underestimated prediction of future cancer incidence may be due at least in part to a low penetrance of cancer registration in LMICs, along with systematic weaknesses in cancer registration that lead to incompleteness in the data, as well as issues related to impaired access to care and low utilization of care such that the formal healthcare sector in LMICs is not encountering the true population incidence.^4,5,7,14,16–18^ This is consistent with the major increase in cancer incidence occurring within the geography that is least equipped to measure it. Moreover, the increase in cancer incidence and mortality within LMICs is diverse and uneven, with rates of increase often most rapid in the lowest income settings, and heterogeneity across regions regarding the histologic makeup of the cancer burden. In some regions, a significant portion of the cancer burden continues to be secondary to infectious diseases such as hepatitis viruses, human papillomavirus, human herpesvirus 8, and human immunodeficiency virus (HIV).^1,5,19,20^

In 1970, only 15% of reported cancer incidence was attributed to LMICs.^21^ This has now increased to an estimated 59%, while LMICs bear approximately 71% of the worldwide burden of mortality due to cancer.^2^ Individuals in LMICs with cancer are considerably more likely to die of their disease, and orders of magnitude more likely to do so without palliation of associated symptoms.^3^ Given the observed trends and causal factors relating to distribution of worldwide cancer burden, it is clear that demand for treatment of cancer will remain or become a leading concern of healthcare systems in every country on the planet for the foreseeable future.^2,3,13^ Although highly developed countries are now seeing a decline in mortality rates associated with cancer, it remains the leading cause of premature death (between the ages of 30 and 69 years) in those countries. Cancer currently ranks as the second, third or fourth leading cause of premature death in most LMICs, after other NCDs, but should be expected to climb in the ranks as these countries follow in the epidemiologic patterns of more developed nations.^1^ Regardless, LMICs already have a greater relative gain to be realized in overall population longevity by preventing premature deaths from cancer than HICs do.^22^ Cancer is a severe and constant threat to public health and economic health in every region of the world through its impacts on rates of disability and death; in HICs, this threat is unrelenting and in LMICs this threat is rapidly worsening.

In context of these demographic and epidemiologic phenomena, experts worldwide have been progressively recognizing cause for alarm, issuing calls to action and dire warnings.^4,7,9,16,17,23,24^ In a 2010 paper marking the creation of the Global Task Force on Expanded Access to Cancer Care and Control in Developing Countries, Farmer et al. described ‘gaping voids in cancer care and control worldwide’ and called the need to address this crisis as ‘an urgent health and ethical priority’.^4^ Cancer in LMICs is a ‘neglected disease’ with ‘severe access limitations’ according to Eniu et al.—an ‘epidemic’ that ‘will become the leading public health issue’ in these nations.^3^ In 2022, Ngwa et al. described the ‘growing cancer crisis’ and pleaded that ‘urgent action is needed’.^5^ These discussions recognize both the current profound disparities in access to cancer care and control around the world, as well as expectation that these disparities will dramatically worsen without decisive intervention.^1–5,7,16,17,22^

Increased awareness of cancer’s growing global threat has coincided with increased awareness of the inadequacy of surgical systems worldwide—recognizing surgery as an especially neglected element of healthcare systems across the developing world.^10,25^ This has major implications on the ability to provide adequate treatment to the growing cancer burden. In 2015, publication of the Lancet Commission’s Global Surgery 2030 report concluded that 5 billion people worldwide lack access to surgical care, whether for benign or oncologic indications, mostly in LMICs.^10^ In the same year, Sullivan et al. published the Lancet Oncology Commission’s Global Cancer Surgery report, which added that about 80% of new cancer cases will need surgery, some of them multiple times, and estimated that in the year 2030 there would be 17.3 million patients requiring surgical procedures for diagnosis, treatment, and/or palliation of cancer worldwide. Of these individuals with cancer, fewer than 25% will be expected to have access to ‘safe, affordable, and timely surgery’. The rate in middle-income countries will be 20%, and just 5% in low-income countries. The report emphasizes the need to prioritize surgery as a core component in plans for both cancer control and universal health coverage (UHC), an objective widely supported by the global health community.^7^

Strengthening surgical systems for cancer treatment requires multiple complex and interconnected investments, including infrastructure, equipment, workforce (not only of surgeons but also of ancillary staff and complementary medical disciplines), and systems for service delivery, financing of services, and management of information.^7,26,27^ Advocates for surgical systems strengthening recognize that training more surgeons is a core requirement, and this includes a need to train more surgeons in their capability to treat cancer with excellent outcomes.^3,7,10,17^ Zafar et al. analyzed the global surgical workforce for cancer and estimated that 9.5 million cancer surgeries were required in 2018, with the ratio of patients needing cancer surgery to the surgical workforce observed to be approximately 10 times higher in LICs than in HICs.^8^ Perera et al. performed a modeling study of the optimal surgical and anesthesia workforce for treating cancer, in which they estimated an increase in annual cancer surgeries needed worldwide from a little over 9 million in 2018 to over 13.8 million surgeries in 2040. Their model indicated that a workforce increase of 25% in HICs, 10% in upper MICs, 67% in lower MICs, and 383% in LICs would be needed to correct current deficiencies, and projected that the demand for cancer surgeons will be further increased by 29% in HICs, 51% in upper MICs, 67% in lower MICs, and 107% in LICs by 2040. The greatest current deficiency and projected growth in demand is in LICs, such that the current workforce of surgeons in those countries would need to multiply by a factor of 9.7 to meet anticipated 2040 demand.^6^ Both studies acknowledged limitations in the data on cancer incidence in resource-constrained settings, and other publications have given higher projections for the increase in needed cancer surgeries.^6,8^ For example, Sullivan et al. suggested the number might be as high as 17.3 million cancer patients with an indication for surgery in the year 2030, with 10 million of those in LMICs.^7^ Similar workforce and access deficiencies exist across many medical specialties, including those that most actively participate in the multidisciplinary management of cancer, such as pathology, radiation oncology, and medical oncology.^3,23,28^ Related to this, surgeons in many LMIC settings take on the management of other oncologic treatments, such as chemotherapy.^3–5,17^

## Unfinished Work in the Landscape of Sustainable Development and Systems Strengthening

The crisis is not new within the history of modern oncology, but it is evolving. Roswell Park—the surgeon who in 1898 was instrumental in founding the cancer research institute that now bears his name—lobbied for public funding based on the proliferating threat of cancer and the ignorance of its causes and treatments.^29^ The second annual report for this institute for the year 1899 included a quote from the Philadelphia Medical Journal, celebrating their work to study cancer: “this pitiless enemy of civilization, which is increasing in such a startling way”.^30^ James Ewing, the pathologist who is credited as the driving force behind the early 20th century revitalization of New York City’s Memorial Hospital as a model for modern cancer hospitals, and for whom the Society of Surgical Oncology (SSO) was originally named, recognized cancer as a public health threat and the need to take a multipronged approach toward its control.^31–33^ The Union for International Cancer Control was founded in 1933 on the premise that the control of cancer was an international scientific priority.^34^ Recognition of the increasing and critical public health threat represented by cancer has been a constant throughout the history of surgical oncology as a profession, and has been the driving force behind the creation of multiple other public and professional societies and institutions.^35^ The sense of nihilism and discouragement that sometimes characterizes current attitudes towards cancer in LMICs mirrors perceptions that were held in HICs just decades ago.^9,17,35^ Tremendous progress has transformed perspectives on cancer over the past century and more within the world’s leading cancer centers, serving to highlight the unfinished work left by past visionaries for us, their heirs. Unfortunately, our efforts contend not only against cancer and constrained resources but also the impacts of conflict and pandemic.^36–40^

The work ahead is more than SWTC can accomplish alone. It will require a consistent, concerted, collaborative engagement with the many stakeholders attached to the issue, recognizing that solutions for worldwide cancer control align with crucial needs and agendas in global health and development. Strengthening systems for surgical treatment of cancer is integral to strengthening surgical systems in general, recognizing a system capable of providing (frequently more complex) cancer operations will typically also be capable of providing treatment for benign disease.^7^ Strengthening surgical systems is also integral to overall healthcare systems strengthening, as one-third of the global burden of disease is surgical, surgical treatments save lives in meaningful and cost-effective ways, and surgical and non-surgical services are complementary and interconnected in functional healthcare systems.^27^ Strengthening surgical services involves support for the entire care continuum, including perioperative care. For instance, the ASOS study showed that patients are twice as likely to die from routine surgery in many countries in sub-Saharan Africa due to deficits in supportive care.^41^ Healthcare systems strengthening is in turn integral to the global development agenda. Underscoring the importance of surgery and cancer control within this construct, the 68th World Health Assembly in 2015 approved resolution WHA 68.15, affirming the need to include surgical and anesthesia services in strengthening healthcare systems to achieve UHC. The 70th World Health Assembly in 2017 approved resolution WHA 70.12, affirming the need for national and global entities to prioritize investment in cancer prevention and control.^27,42–44^

In 2015, the member states of the United Nations unanimously endorsed the Sustainable Development Goals (SDGs), a group of 17 aspirational objectives with subsidiary targets and metrics to guide worldwide development plans leading up to 2030.^45,46^ SDG 3 focuses on ‘Good Health and Well-Being’, encompassing multiple supporting targets. Target 3.4 aims to reduce premature deaths from NCDs such as cancer by one-third, through prevention and treatment, by 2030. Target 3.8 aims to secure UHC, ensuring access to high-quality essential health services with protection from associated financial toxicity.^45,47^ Given our discussion of the rising threat of premature deaths from cancer and the role of surgical treatment in preventing them, achieving targets 3.4 and 3.8 inescapably requires efforts to strengthen systems for the surgical treatment of cancer. Improving equitable access to surgical treatment for cancer also contributes to achieving other SDGs, including SDG 1, ending poverty; SDG 4, ensuring access to education; SDG 5, ensuring gender equality; SDG 9, fostering innovation and infrastructure development; and SDG 10, reducing inequality within and among countries. Yet, while achievement of health targets will support achievement of other SDGs, these targets do not appear to enjoy indirect benefits from investments elsewhere and thus require direct investment.^45,46,48^

The sustainable development paradigm is complex and ambitious. Skeptics observe that as with the millennium development goals that preceded the SDGs, it is unlikely that all targets will be achieved and that there are challenges in prioritizing allocation of scarce resources among these goals. However, the SDGs provide structured acknowledgment of priorities and frontiers for development that are shared among HICs and LMICs, providing a framework for the discussion of public and private investment within which arguments can be made for investments to strengthen surgical systems for cancer. The paradigm also allows recognition that disparities and deficiencies in access to the surgical treatment of cancer are a universal challenge, and attention is needed to correct them in every country, whether an HIC or an LMIC. Barriers to equitable access to surgical treatment of cancer exist for underserved populations in HICs as well as in LMICs, as evaluated by both geographic and demographic distinctions.^6,7,45,46,49,50^ Although they will not be ‘one-size-fits-all’, solutions to these disparities will be fundamentally similar whether employed within HIC or LMIC contexts, with attention needed toward context and adaptation. An understanding of how the priorities for capacity building in global surgical oncology relate to the broader priorities and agendas within global public health and worldwide sustainable development should be central to collaborative efforts between SWTC and the high-level stakeholders positioned to interact with efforts in this area, such as international agencies, national governments and their agencies including ministries of health, non-governmental organizations, and donors. Sustainable development is a paradigm for shared gain, offering prospects of improved geopolitical and economic stability and creating opportunities for enhanced international strategic partnerships built on capacity for trade rather than need for aid. Considering all this, stakeholders with an incentive to invest in strengthening surgical systems for cancer include the governments and institutions of every country on Earth.^40,48,49,51^

Eliminating deficiencies and disparities in global surgical oncology will certainly require significant financial resources. Multiple authors have observed that both cancer control and surgical systems have been neglected by global health initiatives, seemingly under a misconception that cancer is a group of diseases that are too complex and expensive to be treated in resource-constrained settings, and that surgical treatments are likewise unaffordable in these areas.^3–5,7,16,17,29^ However, many of these authors also argue that rather than seeing these challenges as needs the world cannot afford to address, they must be seen as needs the world cannot afford ***not*** to address.^3,4,7^ Sullivan et al. estimated that the failure to meet the needs for surgical treatment of cancer worldwide would correspond to an estimated economic loss of $6.2 trillion over the time period of 2015–2030.^7^ Alkire et al. projected a cumulative gross domestic product (GDP) loss of more than $12.1 trillion for the same time period, caused by failure to provide surgical treatment for cancer, based on a ‘value of lost output’ calculation.^52^ Worldwide economic figures from 2017 suggested the costs of the disease burden of cancer to be an estimated $1.16 trillion per year, while the resources invested on treating cancer were $300 billion.^18^ Countries around the world stand to lose at least 0.5–1.5% of GDP annually to the economic burden of surgically treatable cancers.^7^ Calculating costs associated with surgically treatable cancers on the broader ‘value of a statistical life’ methodology would suggest an annual cost of up to $7.4 trillion per year from death and disability, or a value approaching as much as 10% of annual GDP in some HICs.^7,52^

Regardless of the methodology used to assess the costs, the economic impacts of the failure to surgically treat cancer are clearly very expensive in terms of lost productivity and quality of life, and the differential between the amount of resource invested in the treatment of cancer and the economic losses associated with it suggest ample opportunity to justify dramatic increases in investment.^7,52–54^ Resources directed to healthcare should be seen as wise allocations expected to generate meaningful yields.^7,18,43,52,54^ Investment in healthcare is a driver of broader economic growth, creating demand for jobs, higher education, industrial growth, infrastructure, and innovation; according to the McKinsey Institute, for every dollar invested in health, $2–$4 of economic benefit stands to be gained.^7,18,43,52,54,55^ Even more bullish, the ‘value of additional life-years’ approach would estimate a 9- to 20-fold return on investments in health.^54^ High-level recommendations on resource allocation in global health have increased benchmarks for cancer control, suggesting that LMICs should direct more than two-thirds of their healthcare budgets toward management of NCDs such as cancer, including surgical capacity to treat these diseases.^43^

## So, What is a Surgeon To Do?

A significant number of publications have drawn attention to challenges and needs in the global practice of surgical oncology, and many have laid out broad priorities and strategies. Still, SWTC may feel that opportunities to engage with the issue are limited, intimidating, inaccessible, and/or associated with a low likelihood of professional reward.^3,4,7,9,56,57^ While efforts have been made to propose the roles of surgeons in this area, there remains a need to strengthen the definition of these roles and their importance, to secure increased participation of surgeons, and to commend their contributions.^3,5,7,9,17,56–58^ Included below are specific categories of critical opportunities recognized across multiple assessments of the global oncology landscape.

There is a critical need for SWTC to participate in **advocacy**. This is an opportunity to more proactively and expansively define the position of surgical oncology on the global stage. Surgeons should engage in campaigns to raise awareness among populations and their leadership regarding the concerns discussed in this paper and its cited references. SWTC should advocate for inclusion of surgical services for cancer in the design and implementation of plans for cancer control and for healthcare systems strengthening.^20,59^ Within these efforts, surgeons should collaborate with other oncologic disciplines to draw attention to the importance of prevention, screening, early detection, safe and effective treatment, surveillance, and palliation, as well as the value of multidisciplinary care for cancer and the need for comprehensive systems strengthening to provide it.^23^ SWTC and their professional societies and institutions should also participate along with advocacy groups such as the Global Forum of Cancer Surgeons (GFCS) and the Global Alliance for Surgical, Obstetric, Trauma and Anaesthesia Care (G4 Alliance) to engage with high-level stakeholders to create and direct attention toward effective opportunities for investment.^58,60,61^ When engaged in advocacy efforts, there is an opportunity to emphasize the investment paradigm—resources directed toward these needs are not losses, but are wisely allocated, designed to ***prevent*** further loss, to improve population longevity and productivity, and to contribute to sustainable development and economic growth.^7,18,43,52,55^

Work is also needed in **research**. The community of SWTC has a long tradition of holding in high esteem the surgeon-scientist and recognizing the need for continued research by surgeons not only into the surgical innovations that will advance the field, but into the understanding of the molecular and biochemical structures and behaviors of cancer, and the targets these present for treatment.^31,33,62,63^ While this work is indeed important and more of it needs to be done in diverse settings—especially including LMICs—more surgeon-scientists are also needed who will employ the sciences of sociology, economics, and public health, among others, to overcome barriers to dissemination, implementation, and access to best practices derived from what is learned by other surgeon-scientists in laboratories and leading clinical institutions.^23,43,62–65^ Particularly in low-resource settings, surgeons find their efforts as investigators constrained between lack of funding sources and the demands of high clinical volumes. There is an opportunity to not only continue to advance the science of how to prevent and treat cancer across all settings but to exponentially increase the impact of those discoveries by advancing the science of optimizing the applicability and accessibility to those discoveries’ benefits for everyone on the planet.^63^ There is also an opportunity to recognize and clarify the academic value of all aspects of this work.^3,56,57,63,65^

**Training** of additional workforce for surgical treatment of cancer patients is another critical need. While there is diversity in the training pathways for SWTC around the world, work has been done to suggest core elements of knowledge and skill to be included in curricula for these surgeons.^66–68^ The International Federation of Head and Neck Oncologic Surgeons (IFHNOS) has established a successful Global On-Line Fellowship (GOLF) for training of surgeons around the world in the treatment of head and neck cancers. This program creates opportunities to reach a greater number of trainees while offering them supportive learning structures to enhance their skills and knowledge within a diversity of practice settings in countries around the world; the leaders of this program offer it up as a model to replicate in other disease sites in order to meet global needs.^69^ There is an opportunity for surgeons to support and participate in the concept that training should be the ‘right size’: the optimal content and duration of training for candidates from, in, and for the populations and practice settings where they are needed, and to participate in increasing the capacity across all settings for training SWTC to meet future global workforce needs.^58,66,69,70^

SWTC are also critically needed to participate in efforts for **sustainable development and systems strengthening**. There is an opportunity for surgeons to apply for and administer grants focused on development and capacity building; to create enduring programs and partnerships that enable SWTC to leverage their collective abilities and energies to address critical priorities; and to otherwise offer their own time, knowledge, and skills to enhance capacity in areas of great need. These efforts should focus on collaborative approaches to addressing needs as perceived by all relevant stakeholders, while minimizing waste and disruption and maximizing sustained benefit.^23^ The pitfalls of short-term volunteerism and of projects or programs implemented without adequate stakeholder input should be considered and avoided.^47,71–74^ Multiple examples exist of bilateral or multilateral international partnerships and programs that have had enduring effect; where these have achieved success and contributed sustained benefit, there is an opportunity for SWTC to emulate and expand.^3,4,62,75,76^ Within these efforts, it must be recognized that there is tremendous diversity and heterogeneity in epidemiology and resource allocation not only among countries but sometimes within countries such as India where incidence rates and treatment options vary between regions; strengthening initiatives will need to be context-specific but informed by unified guiding principles.^5,77^

Within the arc of human history, the discoveries that have enabled major improvement in cancer survival are new achievements, contemporary to such fields of development as telecommunications and aviation.^35^ The area of telecommunications in particular is apt for discussion of the challenges associated with meeting the world’s need for cancer control. LMICs have proven the ability to import and adopt later, more streamlined and/or more cost-effective iterations of technologies, to develop innovative applications of these technologies, and to seize opportunities to leapfrog over earlier generations of technologies to find more accessible and scalable solutions.^78^ Both telecommunications and aviation have also combined to make the sharing of knowledge and the development of global collaboration more facile than ever before. A wealth of opportunity exists to explore innovative and collaborative approaches to the challenges discussed in this paper, including exploration and scaling up in applications of information and communication technologies to mitigate disparities in access.^47,79,80^

Access to surgical treatment for cancer is a public health commodity much like several that have been previously recognized and tackled, many with achievement of significant success. There is an opportunity to learn from past successes and to approach the problem of inadequate access to surgical treatment of cancer with techniques analogous to those taken to improve global access to various vaccines, mosquito nets, and antiretroviral drugs for HIV.^3,4^ The history of HIV initiatives in LMICs is instructive; critics said that achieving global access to HIV therapies would be too complicated and too expensive, but programs for this have been quite effective.^3,4^ Successes will be achieved by breaking the issue into component parts and taking systems-based approaches to addressing them. Successes will also be achieved by leveraging the political will and resources of multiple stakeholders. There is a wealth of opportunity to be part of these efforts and part of their successes.

## Conclusion

While the work of SWTC has achieved major advancements through the history of modern oncology, the global landscape of surgical oncology is fraught with deficiency and disparity, with critical needs for capacity building and sustainable development. The worldwide burden of neoplastic disease is increasing, and capacity for cancer control requires augmentation and attention to correcting disparity in every country on the planet. While this is true for HICs, LMICs represent the majority of both the current unmet need and the expected future increase in need. Opportunities abound for surgeons to engage in combating disparities on a global scale, as well as in their own local and national contexts. SWTC around the world are needed—both as individuals and through their collective participation in professional societies and other entities, institutions, and alliances—to engage collaboratively as leaders in advocacy, research, training, and sustainable development to create stronger systems for surgical treatment of cancer. These efforts must be bold and ambitious if we are to avoid a future in which deficiency and disparity are further exacerbated and entrenched.
